# An Investigative Study on the Progress of Nanoclay-Reinforced Polymers: Preparation, Properties, and Applications: A Review

**DOI:** 10.3390/polym13244401

**Published:** 2021-12-16

**Authors:** Dalia E. Abulyazied, Antoaneta Ene

**Affiliations:** 1 Department of Physics, Faculty of Science, University of Tabuk, Tabuk 71491, Saudi Arabia; daliaabulyazied@outlook.com; 2 Department of Petrochemical, Egyptian Petroleum Research Institute (EPRI), Cairo 11727, Egypt; 3 INPOLDE Research Center, Department of Chemistry, Physics and Environment, Faculty of Sciences and Environment, Dunarea de Jos University of Galati, 47 Domneasca Street, 800008 Galati, Romania

**Keywords:** polymer, clay, nanoclay, nanocomposites, preparation, mechanical, thermal, applications

## Abstract

Nanoclay-reinforced polymers have attracted considerable universal attention in academic and industrial research due to their outstanding properties and their ever-expanding utilization in diversified applications. In that regard, in the present review, the structure of layered silicate clay, as well as procedures for clay material modification, are outlined. We also discuss the general characterization techniques, synthesis methods, and various properties of polymer–clay nanocomposites (PCNs), and some examples likewise are depicted from the scientific literature. The study’s primary goal is to provide an up-to-date survey of polymer–clay nanocomposites and their specific applications in industries such as automotive, flame-retardant, and biomedical applications, coating, and packaging.

## 1. Introduction

**Figure 1:**
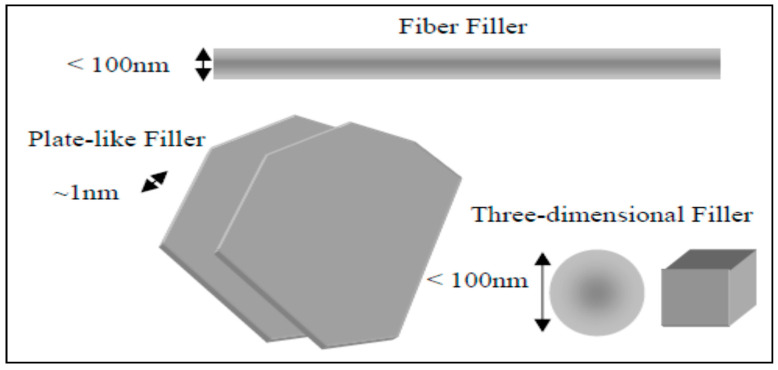
Schematic illustration of nanoscale fillers.

Polymer nanocomposites are a significant class of inorganic–organic nanocomposites, in which inorganic nanometer materials are distributed in the matrix of the organic polymer. The present tendency in producing innovative nanocomposites materials is exemplified by these polymer nanocomposites [1,2,3]. The interface between the filler and the polymer matrix in the nanocomposites constitutes a larger area than in ordinary composites based on micrometer-sized fillers and therefore impacts the properties of the nanocomposites to a much greater degree, even with minimum filler [4,5]. Nanoscale materials have a variety of shapes and sizes; we have divided them into three groups [6] (Figure 1). One-dimension (1D) nanoplatelets, such as nanoclay [7] and nanographene [8,9], are layered particles having a diameter of ~1 nm and a length of hundreds to thousands of nanometers. Fillers in the second group composed of fiber or tubes have a thickness of at least 100 nm and an aspect ratio lea than 100, such as carbon nanofibers and nanotubes [10]. Three-dimensional (3D) nanofillers, such as spherical silica [11,12], nanoalumina, and nanotitanium oxide, are approximately equiaxed particles with the greatest dimension of less than 100 nm. Many nanofillers (carbon nanotubes, nanosilica, nanocellulose, and graphene) have been examined for their reinforcing ability; however, easily available clay and low cost have been considerably examined as nanofillers for the different polymers, which lead to the great enhancement of their properties at very low filler loading [13,14,15,16,17].

These polymer–clay nanocomposites have several economic benefits, including lower weight due to the lower clay content (2–5 wt. percent), which is industrially appealing. Improved mechanical properties, such as stiffness and strength, better permeation-barrier behaviors as a result of the reduced direct motion of oxygen and water molecules, and greater thermal properties, such as flame retardancy and heat distortion temperature (HDT). These incredible qualities make polymer–clay nanocomposites attractive materials for everything, from foods and electronics packaging to durable, heat-tolerant vehicle parts. Toyota research group first introduced polymer–clay nanocomposites by the end of 1985, when they successfully prepared nylon 6 clay nanocomposites to possess outstanding heat distortion temperature, strength, modulus, gas and water barrier properties when compared with neat nylon 6 [18]. This study is regarded as the primary inducement for the researcher in this field, by investigating the influence of nanoclay additions on the structure and properties of several polymer matrices for various applications, for example, but not limited to, polystyrene (PS) [19], polycarbonate (PC) [20], polyamide-6 (PA-6) [21], polyacrylamide (PAA) [22], polyurethane [21], and polyvinyl alcohol [23].

Clay is made up of layered silicates (aluminum phyllosilicates) that contain metal oxides including alkali earth metals, alkali metals, calcium, and other metal oxides, in addition to organic elements in the smallest possible quantity [13,24]. Clay ingredients are split into two types based on how the alternating sheets of “SiO_2_” and “AlO_6_” units are arranged: (i) 2:1 clay (smectite and vermiculite) and (ii) 1:1 clay (kaolinite). The high aspect ratio of smectite clay minerals is due to their unique intercalation–exfoliation action, which makes them particularly significant and potent as reinforcing fillers for polymers. It belongs to a group of five major members, the most well known of which is montmorillonite, which has a plethora of uses. Other clay material, such as the 1:1 structure, is not typically employed in polymer nanocomposites because they lack effective intercalation and exfoliation properties or are difficult to layer separately [25]. The layers are tightly packed together due to their increased charge density, making hydration of interlayer positive ions or penetration of polymer chain into the layers hard or impossible [1].

The goal of this paper is to outline recent scientific and technological progress in the field of polymer–clay nanocomposite, in order to gain valuable insight into how superior-performing nanocomposites are developed. This is accomplished by reviewing the various preparation methods, characterizing these materials, and classifying them into different formulations, as well as reviewing their excellent properties and various applications in the industry. Finally, prospects and aspirations for the use of this material are articulated.

## 2. Structure of Clay (2:1 Phyllosilicate)

**Figure 2:**
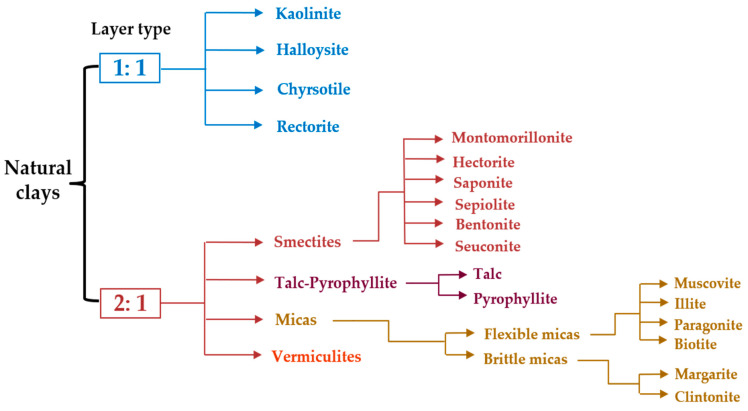
Classification of clays.

**Figure 3:**
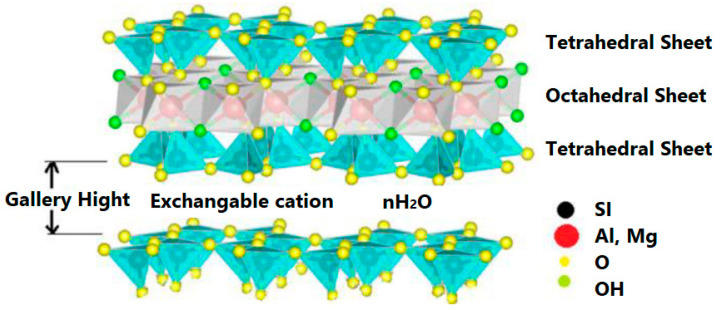
Structure of 2:1 layered silicate.

For a better understanding, Figure 2 depicts the broad classification of clay materials [1,13,26]. Bentonite or montmorillonite clay is mainly composed of 1 nm thin layers of 2:1 silicate with a center alumina octahedral sheet fused between two outer tetrahedral sheets of silica (by sharing oxygen atoms) and the presence of sodium and calcium ions inside the gallery (Figure 3) [27,28].

## 3. Modification of Clay Particles

**Figure 4:**
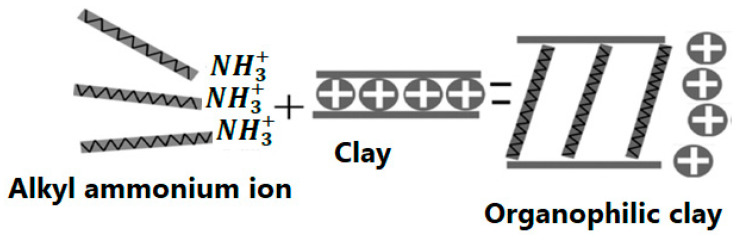
Schematic representation of an ion-exchange reaction.

**Figure 5:**
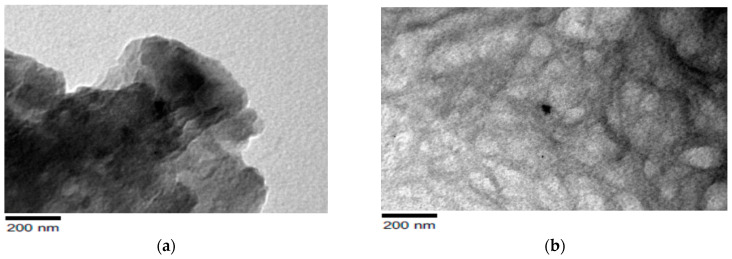
TEM images of organoclay (**a**) before modification and (**b**) after modification with octadecyl amine, reprinted from [7].

The covalent bonds that occur between the interlayers of clay layers, along with the hydrophilic nature of the clays, make them unsuitable to reacting and dispersing with most polymer matrices. Furthermore, electrostatic forces closely hold the stacks of clay layers together. Clay particles are modified before being dispersed in polymer matrices to solve this challenge. Essentially, the distance between the sheets of clay layered silicates is increased during the modification process by surfactants intercalation. It is feasible to introduce hydrophobic character into clay minerals by such changes, allowing the dispersion of modified clay very fine in polymer matrices. For the modification of clay structure, there are two basic techniques: (i) physical modification and (ii) chemical modification. Only the adsorption of modifying chemicals on the clay surface occurs in the physical modification process. This approach does not modify the clay particles, but it does help to marginally improve the characteristic of the produced polymer composites. The small amendment in characteristics could be attributable to the fact that the modifying chemicals and clay have only weak van der Waals forces. However, chemical modification aids in the creation of chemical reactions between the layered silicates and the modifier. Furthermore, cationic or anionic functional groups are used in the ion exchange process to modify the molecules. As a result, the capability of clay to disperse in polymer matrices is improved. The improved mechanical, thermal, optical, and barrier properties of polymer/nanoclay composite systems are due to the nano-thick layers existing in the clay material, as well as the larger surface area and higher aspect ratio exhibited by clay particles when distributed in polymeric composites [29,30,31]. The alteration of clay particles is depicted schematically in the diagram below (Figure 4) [31]. TEM images of clay bentonite before and after modification with the quaternary ammonium salt of octadecylamine are shown in Figure 5 [7].

## 4. Processing of Clay/Polymer Nanocomposites

### 4.1. Solution Blending Method

**Figure 6:**
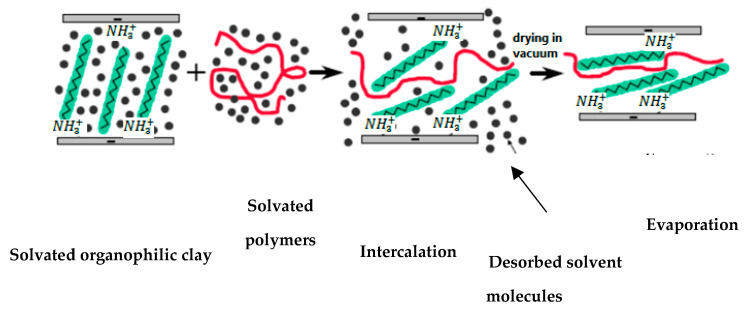
Solution blending technique for the preparation of polymer/clay nanocomposites.

Typically, the polymer is dissolved in a particular solvent before being used in this technique. At the same time, clay is dispersed separately in the same solvent. Following that, the mixture of clay and solvent is poured into the polymer solution. Finally, the polymer and clay mixture is homogenized for a length of time; then, they are cast on a Peter dish for evaporation of the solvent [32,33,34]. The processes in the solution blending method are depicted schematically in the diagram below (Figure 6).

### 4.2. Melt Blending Method

**Figure 7:**
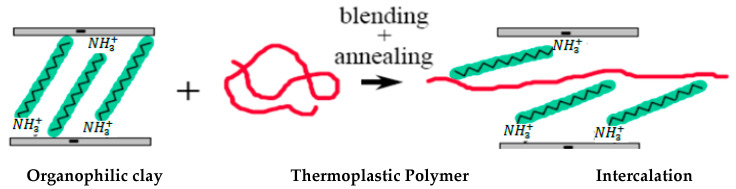
Melt blending technique for the preparation of polymer/clay nanocomposites.

In comparison with the solution blending approach, this method provides superior compatibility between the layered silicates and the polymer. The melt blending technique is used to directly reinforce polymer with clay particles. The polymer and clay mix is annealed in this technique, which is commonly carried out at temperatures over the polymer’s melting point [35,36,37]. The processes in the melt blending procedure are depicted schematically in the diagram below (Figure 7).

### 4.3. In Situ Polymerization Method

**Figure 8:**
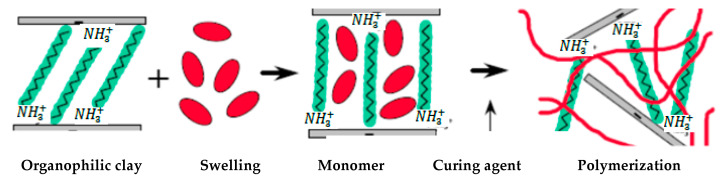
In Situ techniques for the preparation of polymer/clay nanocomposites.

In this method, an initiation step precedes a series of polymerization processes, resulting in the development of a hybrid between polymer molecules and organoclay. Initially, the organo-modified clay is swelled within a liquid monomer or monomer solution, as seen in Figure 8, and subsequently, monomer dispersion occurs into the clay spacing gallery. After that, the polymer molecule is produced via the polymerization process [38]. This polymerization can be triggered by using a source of radiation or thermally, with a suitable catalyst or initiator added before the clay layers expand via an ion exchange process. Immediately after the end of the polymerization mechanism, a nanocomposite containing polymer molecules tethered to nanoclay is formed [39,40].

## 5. Structures of the Nanocomposites PCN

**Figure 9:**
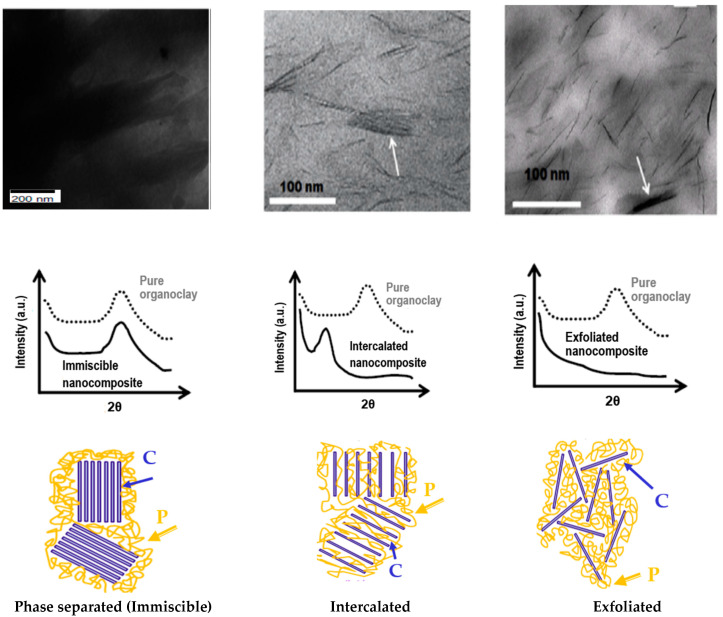
Illustration of various structures of organoclays (C) with polymers (P) (**bottom**) and their corresponding WXRD (**middle**) and TEM images [7,26] (**upper**).

The level of intercalation and exfoliation of the clay particle is highly influenced by the clay type, polymer, organic modifier, and the polymerization method. Three forms of polymer/clay composite configurations are possible based on the level of variation in the reaction between the layered silicates and polymer [13,26], as discussed below and illustrated in the diagram from Figure 9, which contains transmission electron microscopy (TEM) images [13,26], and wide-angle X-ray diffraction (WXRD) patterns of the structures of polymer/organoclay nanocomposites (phase-separated, intercalated, exfoliated).

### 5.1. Phase-Separated Structure

A phase-separated composite is formed when the polymer matrix is improbable to enter and disperse into the spacing between clay layers; this is due to the incompatibility of clay particles and polymers, which results in a weak interaction between the two phases. As a consequence, the spacing between layers of clay is negligible, because the layers of clay are still stacked together and take shape of aggregation around the polymeric matrix. As a result, it functions similarly to a conventional microcomposite, needing a high clay volume fraction to achieve considerable improvements in physical properties [41,42].

### 5.2. Intercalated Structure

When the polymer matrix is intercalated into the interlayer spacing between the clay during the synthesis of a PCN, an intercalated structure is formed causing an increase in this interlayer spacing [43]. It should be emphasized that the periodic array of the clay layers persists in a well-ordered stack between the layers of the clay and polymer matrix [44]. When the same amount of clay was added, this structure had better characteristics than the phase-separated composite.

### 5.3. Exfoliated Structure

Complete disintegration of clay layers as individual sheets results in a delaminated–exfoliated structure, which is distributed well in the polymer matrix. Most of the properties (e.g., thermal, mechanical, and barrier) of the resulting polymer nanoclay composite are expected to increase due to the homogenous distribution of totally exfoliated clay layers into the polymer. In actual conditions, however, obtaining total exfoliation is a difficult undertaking [30]. This structure, as compared with an intercalated structure, provides the most beneficial enhancement in the resulting polymer nanocomposites characteristics due to the high aspect ratio and intense surface reaction of the clay particles with the polymer chains [45,46,47].

The exfoliation arrangement is of specific importance since it provides the highest interaction between the polymer and the modified clay by allowing the polymer to access the entire surface of the silicate layers, therefore resulting in the greatest variations in different physical properties. Nevertheless, there is still some debate about whether entirely exfoliated layered silicates occur in the system of polymer–clay nanocomposites, which is confirmed by a significant proportion of polymer nanocomposites in the literature were found to have intercalated or mixed intercalated–exfoliated nanostructures [33]. This is due to the fact that the silicate layers are highly anisotropic, with side sizes varying from 100 to 1000 nm, and cannot be placed randomly in the polymer matrix even when detached by large spacing [43,48].

## 6. Morphological Characterization of PCN

X-ray diffraction (XRD) and transmission electron microscopy (TEM) are two complementary techniques commonly employed to analyze the structures of organically modified clay and PCN [33,34,35,36]. XRD is most typically employed to explore the nanocomposite morphology because it is easy and accessible to use. Using Bragg’s law, this technique calculates the gaps between the interlayer spacing of the clay: 2dsinθ=nλ, where θ is the observed diffraction angle or incident angle, λ is the wavelength of the X radiation used in the diffraction experiment, and *d* is the interlayer distance between layered silicates [49,50,51].

**Figure 10:**
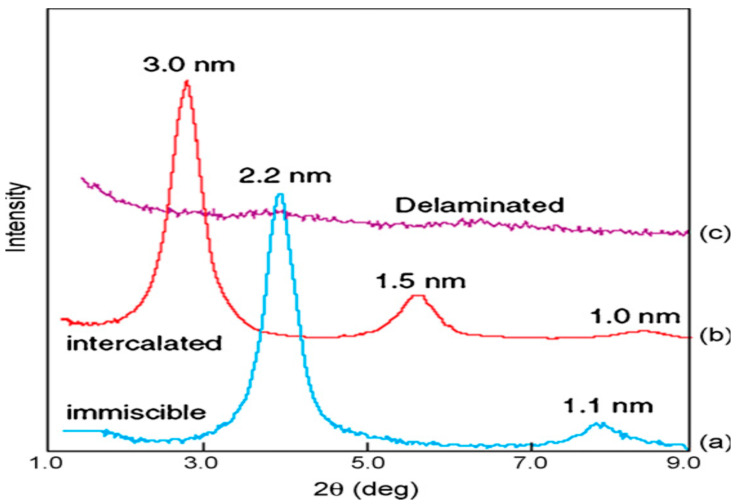
WXRD patterns for polymer/layered silicates: (**a**) polyethylene + organoclay → phase-separated composites; (**b**) polystyrene + nanoclay → intercalated composite; (**c**) siloxane + nanoclay → exfoliated composite [43].

Different structures of polymer–clay nanocomposites (phase separated, intercalated, and exfoliated) can be detected by tracking the intensity, position, and, shape of interlayer spacing diffraction peaks from dispersed silicate layers [50]. The structure of the silicate is unaffected in phase-separated polymer–organoclay mixes, and hence, the features of the organoclay basal reflections remain unchanged. The intercalated structure of polymer-layered silicate, on the other hand, causes expanding of the d-spacing in comparison with the d-spacing of the used modified clay, resulting in shifted diffraction peak to a smaller angle, as Bragg’s law states. The increased interlayer spacing in intercalated nanocomposites implies that the polymer has penetrated the separation and expanded the layers, but the repeating layered structure has been preserved. However, exfoliated structures have substantial layer separation, which interrupts the coherent layer stacking, and no peaks can be observed in the diffraction pattern with the WXRD technique (2θ > 1°) (Figure 10) [43]. The disappearance of the peaks is either due to extreme separation that occurred in the clay layers (i.e., surpassing 8 nm if the exfoliated structure is well ordered) or due to the lack of order in the nanocomposite [52].

XRD provides a reliable technique for establishing the distance between the silicate layers of the clay before modification and for intercalated polymer nanoclay composites (~1–4 nm). However, it is imperfect for measuring delaminated and exfoliated PCN, as it does not produce a peak. In particular, in circumstances where no peak is seen, the lack of peak may be misunderstood. The XRD patterns of layered silicates can be influenced by a variety of factors, including the concentration and order of the layer silicates. Samples with poorly ordered layered silicates, for example, will not yield a diffraction peak, and this is the proper inference from the data. It is not the technique defect if an inaccurate conclusion is reached about the PCN being exfoliated, while it is, in fact, very disordered. As a result, the lack of an XRD peak just indicates that no peak was seen; it does not establish or contradict the presence of exfoliated clay layers in the nanocomposite [53].

**Figure 11:**
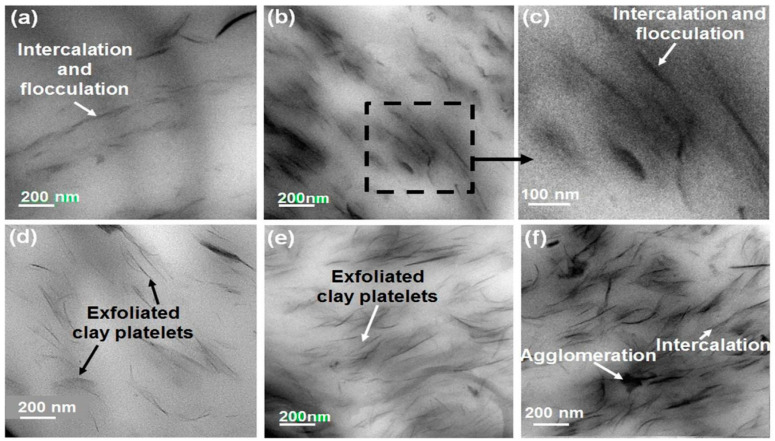
TEM images showing dispersion of clay platelets in CPN: (**a**) PU-CA-1; (**b**,**c**) PU-CA-3; (**d**) PU-C30B-1; (**e**) PU-C30B-3; (**f**) PU-C30B-5 [55].

In comparison, TEM provides a qualitative comprehension of the interior structure and can supply information on morphology and defect structures in real time, in a localized area [53,54]. The clay layers are made of heavy atoms such as Al, Si, and O, while the polymer composed of light elements such as C and H, as well as the spacing gallery, contains light atoms such as Na and Mg, so the layered silicates appear in the TEM images as dark lines, while the polymer or the gallery appear brighter. As a result, the spacing between darker line intersections in TEM images can reflect the distance between the clay layers and the state of dispersion qualitative manner. Thus, utilizing the TEM approach, the morphology of the PCN may be determined clearly, including the state of dispersion and imperfections, as well as either intercalated or exfoliated clay [43,53]. The TEM images of polyurethane nanoclay composites are shown in Figure 11 [55].

## 7. Properties of PCN

The goal of incorporating layered silicates into different polymers is to enhance the polymer properties and create PCN with desirable qualities for specific applications. Nanoclays can provide significant and changeable enhanced properties at a quite low volume fraction, which helps to preserve more of the pristine beneficial properties of the polymer. The ultimate properties of polymer–clay nanocomposite are affected by the type and properties of the constituents, as well as the processing method and conditions [43].

### 7.1. Mechanical Properties

Generally, the primary purpose of incorporating inorganic nanoparticles to polymer matrix is the enhancement of mechanical properties; therefore, the inorganic nanoparticles are referred to as reinforcing reagents. The improvement procedure is based on high stiffness, higher strain and, higher modulus of the inorganic nanoparticles. The majority of studies show the tensile modulus of polymeric nanocomposites made using modified organoclay has been significantly enhanced, especially with increasing the loading of the organoclay [43,56]. Nevertheless, in some cases, Young’s modulus decreased, because fully exfoliated structures are changed to partially exfoliated–intercalated structures when the volume fraction of organoclay surpasses the threshold limit value [50,57,58]. In the 1980s, the Toyota research group was the first to report the reinforcement of the nylon matrix with 4.7 wt% of clay, which can significantly improve the mechanical properties of nylon. They created a nylon–clay hybrid (NCH) with better mechanical and thermal properties than unfilled nylon, including a higher modulus, increased strength, and a lower heat distortion temperature [59]. Table 1 displays an overview of Young’s modulus of polymer–clay nanocomposite experiments that have been undertaken for different polymer matrices [60,61,62,63,64,65,66,67,68].

Any parameter that influences the degree of intercalation and exfoliation, similar to modulus, has a significant effect on the tensile strength of nanocomposites. Another impact of nanoclay filler on nanocomposites’ mechanical characteristics is the elongation at break value, which is influenced by the interfacial reaction between the polymer and layered silicates. Burmistr et al. [69] reported a comparative study of mechanical properties such as tensile strength, Young’s modulus, and elongation at break (%) for polypropylene (PP), polystyrene (PS), and polyamide (PA) matrices reinforced with organo-modified clay, results of which are shown in Table 2.

### 7.2. Thermal Properties

**Figure 12:**
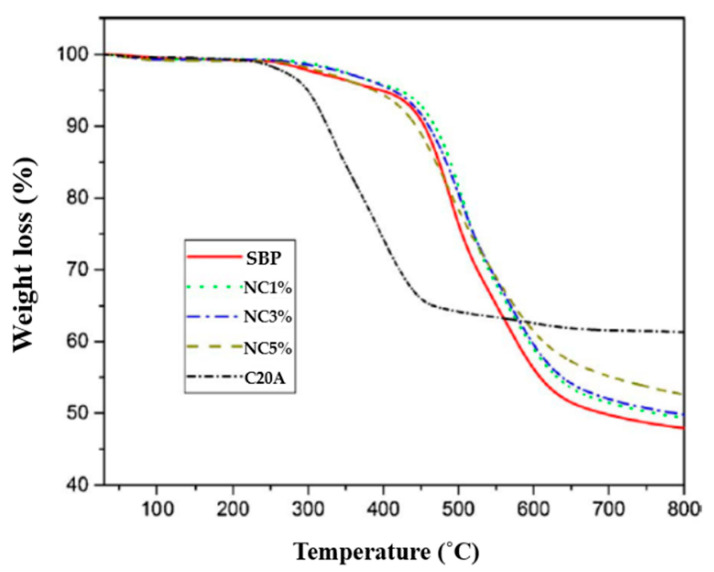
TGA of neat SBP, organoclay, and nanocomposites.

The thermogravimetric analysis is commonly used to determine the thermal stability of polymeric composites (TGAs). The weight loss as a function of temperature due to the production of volatile compounds during high-temperature degradation is measured. Non-oxidative degradation happens when the samples are heated in an inert gas flow, whereas oxidative degradation occurs when the materials are heated in air or oxygen. Clay addition into the polymer matrix was reported to improve thermal stability in general [70,71,72,73]. The increased thermal stability of PMMA–MMT nanocomposite was initially reported by Blumstein (1965), where PMMA–MMT nanocomposites have a 40–50 °C greater decomposition temperature, according to its TGA. The thermal stability of a polystyrene–clay nanocomposite was reported by Vyazovkin et al. [74]. When compared with pure PS, polystyrene nanoclay composites have a 30–40 °C greater degradation temperature than neat PS. Other researchers have found that the type of clay modifier used can influence the thermal stability of modified organoclay and their polymer nanocomposites [39,75,76,77]. The thermal properties of solvent-based polyamide-imide (SBP)–clay nanocomposites are shown in Figure 12, and the starting temperature of thermal degradation and temperatures corresponding to 5 and 10% weight loss are illustrated in Table 3 [78].

**Figure 13:**
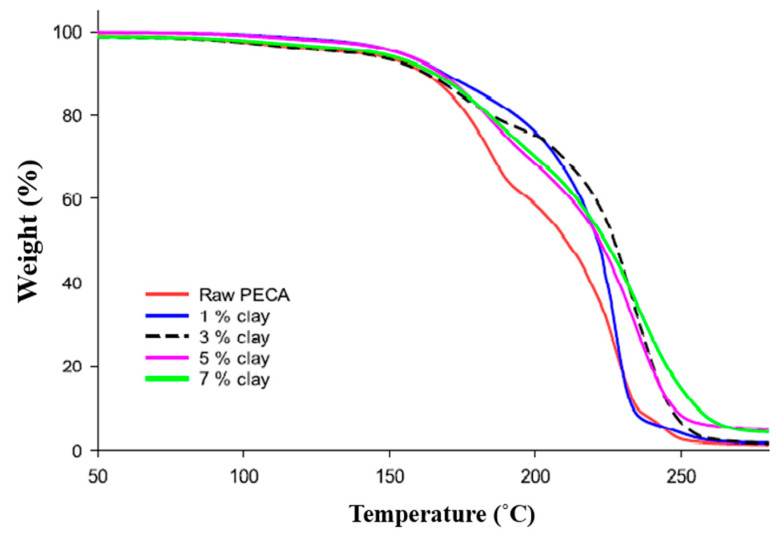
TGA of pure PECA and PECA–modified Mt composites with different wt% [79].

The TGA thermogram of pure poly ethyl cyanoacrylate PECA and PECA–montmorillonite MMT nanocomposites, with various weight percent 1, 3, 5, and 7% of the modified MMT, is shown in Figure 13 [79]. All samples had their decomposition temperatures determined at 10 and 50 percent mass loss (T_10_ and T_50_), and the char residues were estimated using the TGA curve and given in Table 4. At T_10_, all composites decomposed at 9, 2, 6, and 7 °C higher than pristine PECA for 1, 3, 5, and 7 percent (wt./wt.) composites, respectively. At T_50_, all composites showed higher decomposition temperatures than pure PECA by 11, 17, 12, and 14 °C, respectively, for 1, 3, 5, and 7% composites. At 350 °C, PECA has a smaller residue mass than all produced composites, and the residual mass grew at the level of MMT added increased [79].

### 7.3. Gas Barrier Properties

**Figure 14:**
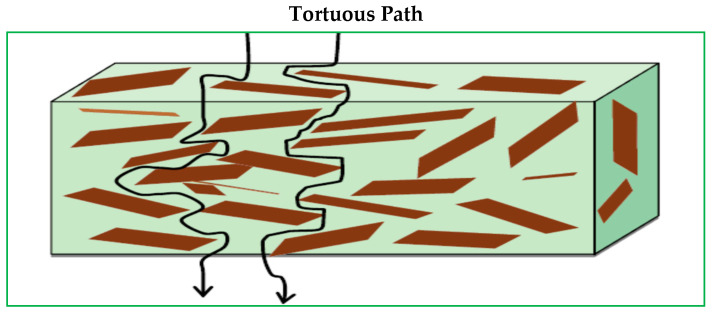
Schematic representation of the mechanism tortuous of improved barrier mechanism by the addition of clay platelets.

The drastic enhancement of polymer barrier qualities is one of the most significant impacts of clays on polymer matrix properties [80,81]. Clay sheets are impermeable by nature. Clays improve polymer barrier characteristics by generating a maze or convoluted path that slows gas molecule migration through the polymer matrix (Figure 14) [81]. The degree of tortuousness formed by layered silicates in the propagation path of their molecules through the polymer matrix determines the degree of barrier enhancement. The tortuous factor is defined by the proportion of the actual distance traveled by a diffusive molecule to the shortest distance traveled by a diffusive molecule (thickness of nanocomposites film). The aspect ratio of clay spread in the polymer matrix has an impact on gas barrier properties. More barrier improvement in the polymer is caused by increasing the lateral length of the layered silicates in addition to the degree of intercalation or exfoliation. The barrier capabilities of PCN versus the transport of gases and vapors have been described in numerous studies [41,82,83,84,85]. Table 5 summarizes the findings of several investigations on the water vapor permeability of polymer–clay nanocomposites [86,87,88,89,90,91,92].

### 7.4. Non-Flammability

Clay particles are layered silicates with a chemical structure that makes them completely fire resistant. Nanoclay has this characteristic, which makes it useful in the manufacture of materials in which non-flammability is more significant. These nanoclays are used as reinforcement in rubbers and other polymers to make them less flammable [28]. In addition, when compared with traditional procedures, other advantages such as modest filler loading are sufficient to accomplish flame retardancy. These non-halogenated flame retardant additives are made out of polymer–clay nanocomposites [93]. A flame retardant nanocomposite was made by intercalation process using common smectite clay montmorillonite and was called flame retardant hide powder. To make flame-retardant leather, this hide powder was mixed with animal skins. Furthermore, leather’s thermal stability was improved [94]. Clay minerals are effective flame retardants in a variety of polymer materials, particularly leather, making them appropriate for the creation of furnishing material in the aeronautic industry [95].

One of the most useful bench-scale approaches for characterizing the flammability properties of diverse clay-based polymer nanocomposites is the cone calorimeter test. Two essential metrics for evaluating fire safety are heat release rate HRR and peak heat release PHRR. HRR is regarded as the fire’s driving force, whereas PHRR denotes the location in a fire where heat is most likely to spread or ignite nearby items [28].

## 8. Applications of PCNs

### 8.1. Applications in Automotive Field

**Figure 15:**
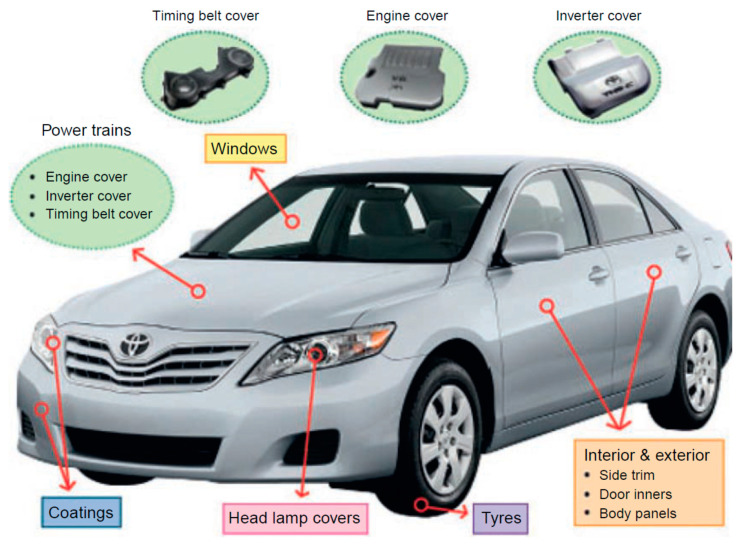
The most essential parts of an automobile where nanocomposites are used.

Fuel efficiency and low emission are among the international objectives for PNCs based on clay nanoparticles as filler in the automotive industry, as they have low cost, high performance, and lighter weight. Toyota Motors pioneered the use of these nylon 6 clay nanocomposites in the early 1990s. The timing-belt cover was created by integrating organo-modified nanoclay in polyamide 6, as the polymer matrix was the first commercial product of polymer–clay nanocomposites. A timing belt is a part of an internal combustion engine that has the function of controlling the timing of the engine’s valves. This timing-belt cover performed admirably in comparison with plastics, with good stiffness, excellent heat stability, and lack of wrapping. As a result, the vehicle’s weight was drastically reduced [96]. Unitika Co. used nylon 6 nanocomposites for engine covers on Mitsubishi GDI engines in approximately the same years that Toyota introduced CPN [97]. Over the years, significant progress was made in the industry, and more vehicle companies began to use PCNs. External vehicle body parts manufactured from common thermoplastic olefin (TPO) combined with nanoclay, for example, were introduced by GM and partners Basell, Southern Clay Products, and Blackhawk Automotive Plastics. This TPO nanocomposite with a minimum nanoclay weight percent (2.5 wt.%) was as rigid as, and significantly lighter than, vehicle parts filled with 10 times the quantity of unmodified clay [98]. The most essential parts of automobiles where polymer–clay nanocomposites are utilized are shown in Figure 15 [99].

### 8.2. Applications in Packaging

The food packaging sector is very interested in the increased barrier properties of nanoclay-reinforced polymers. Advances in barrier properties to water vapor, various gases such as nitrogen, oxygen, and carbon dioxide, and aromatic compounds have recently been researched for PNCs, in addition to enhanced thermal and mechanical properties. Therefore, they are rapidly displacing neat polymers in food packaging. These neat polymers are impermeable and harmful to the environment. High-barrier polyamide nanoclay is manufactured by Mitsubishi and Nanocor under the trade name Imperm^®^ N. When compared with pristine PET, a 1000 times decrease in oxygen transfer rate was recorded in a nanoclay PET bottle [100]. Additionally, under the trade name nano seal, NanoPack Inc. was successful in producing a water-based coat consisting of nano-vermiculite polyvinyl alcohol nanocomposites. Furthermore, nylon 6/nanoclay nanocomposites with improved barrier, mechanical, and thermal properties have been developed and are used in cosmetics, food, medical, and electronics packaging [98].

### 8.3. Flame Retardant Applications

The effectiveness of layered silicates as flame retardants is based on their ability to minimize the content of flame retardant substances while also reducing the degradation of physical and mechanical qualities at the same level as flame retardants. PCN has been used in flame retardant applications in a variety of fields, including the leather industry, making it appropriate for the manufacture of upholstery material in the aerospace industry [95]. In addition, wires and cables with flame resistance and retardancy were obtained by combining organo-modified clay with standard halogenated or non-halogenated flame retardants [101,102,103].

### 8.4. Drug Delivery Applications

The applications of polymer–clay nanocomposites are rapidly gaining traction in medicine and biological items such as injured or lost organs and tissues. Tissue engineering is one of the most important fields that use PNCs to recover missing organs. As pore geometry, pore membrane morphology, and connection between pores of nanostructured materials are critical for growth, cell implantation, emigration, mass transit, and tissue formation, these materials have been proven to be useful in biomedical applications [104,105]. Nanoclay-reinforced polymers are also used in drug delivery systems, which is a major medical application. The design of a drug delivery system includes loading a specific concentration of medication into a specified delivery system and releasing the medicine at the desired pace to the target site. Many efforts have been made to manage the distribution and release of medicine for Alzheimer’s disease by adsorption or intercalation of medicines in montmorillonite clay [106]. 

### 8.5. Other Applications

The enhanced physical and chemical properties of polymer–clay nanocomposites (PCN) made them have more diverse applications, including water treatment. Due to the ease of preparation, effective cation exchange process, stability, high aspect ratio, and minimum toxic effects [107], PCNs possess a high fluctuation and adsorption ability and an incredible life cycle for water purification [108]. Several researchers have developed different PCNs for the removal of different pollutants from hydrous solutions, and they have been demonstrated to be beneficial in the removal of organic and inorganic pollutants [109,110,111,112,113].

PCNs can also be used in Lithium-ion batteries (LIBs); due to their compact size, higher values of energy density, and stable cycling quality, rechargeable LIBs are regarded as one of the most efficient and convenient systems for energy storage in mobile technologies and vehicles operated by electricity.

Self-supported polymer gel electrolytes (PGEs) based on poly (vinylidene fluoride) (PVDF)–clay nanocomposite, with 0–4 wt.% loading of the modified clay, has been fabricated, and their electrochemical properties in lithium-ion batteries were investigated. The evaluation of PGE performance in Li/LiMn_2_O_4_ cells illustrates the accessibility of PVDF-modified clay nanocomposites-based PGEs for lithium-ion battery applications [114].

Young Min Jeon et al. fabricated a UV-crosslinked electrolyte made from a polymer–clay composite composed of ethoxylated trimethylolpropane triacrylate solvated polyvinylidene fluoride-co-hexafluoropropylene (ETPTA/PVDF–HFP) as a matrix and Cloisite20A (modified clay) and their electrochemical properties in lithium-ion batteries were investigated. They found that the organoclay contributes significantly in improving ionic conductivity and increasing the transference number of lithium cations, leading to the superior performance of electrochemical battery charge and discharge [115].

## 9. Conclusions and Future Perspectives

Of the many acclaimed technology products, polymer–clay nanocomposites are ones that meet expectations. Polymer–clay nanocomposites have superior mechanical, thermal, barrier, and flame-retardant properties, compared with micro- or macrocomposites. For this reason, polymer–clay nanocomposites have shown a widespread presence in a variety of application areas. Polymer nanocomposites, for a variety of applications, can be created by selecting the appropriate matrix, modified organoclay, and synthetic methods. This study discussed recent advancements in new polymer–nanoclay composites, with important applications in a variety of industries, including the automotive field, packaging, flame-retardant, and drug delivery applications.

This paper provided an overview of the different classes of natural clay and the structure of smectite clay, as well as the modification routes of layered silicates. In addition, the methods of preparation polymer organoclay nanocomposites and their characterization techniques were elucidated. Furthermore, the different properties of polymer nanoclay composites such as mechanical, thermal, barrier, and flame retardant properties were outlined. Finally, the important applications of these materials were discussed in detail.

However, although polymer–clay nanocomposites have many important uses in various industrial fields, there is a large number of technical and economic obstacles to ubiquitous commoditization. These include impact resistance, complicated formulation relationships, and methods for accomplishing and measuring the dispersion and exfoliation of nanoclay in polymer matrices. Another restriction to bringing out novel nanocomposites technologies is the investment in ultramodern devices and the expansion of the academic research groups.

Future predictions included in the review involve the application of this nanotechnology to different kinds of polymer systems and various routes of clay modifications, which would almost certainly necessitate the implementation of novel strategies.

## Figures and Tables

**Table 1:** Young’s modulus for different polymer–clay nanocomposites.

Polymer Type	Pure Polymer Modulus, GPa	Nano-Clay Wt.%	PCN Modulus GPa	Ref.
Polyester	2.87	5	3.79	[60]
Polyamide	2.45	7	3.46	[61]
Nylon 6	1.2	5	2.43	[62]
Epoxy	2.05	5	3	[63]
Polypropylene	1.76	7	2.7	[64]
Polyurethane	0.025	13	0.45	[65]
Polycarbonate	2.3	4	3	[66]
Polylactic acid	3.6	5	4.8	[66]
Polyvinyl chloride	0.209	4	0.54	[67]
LDPE	1.05	5	1.09	[68]

**Table 2:** Mechanical properties of some polymer–clay nanocomposite.

Polymer	Content (Wt.%)	Unmodified Clay			Organo-Clay		
		Tensile Strength (MPa)	Elongation at Break (%)	Young’s Modulus (MPa)	Tensile Strength (MPa)	Elongation at Break (%)	Young’s Modulus (MPa)
Polyamide 6	0	30.86	28.75	1.07	30.86	28.75	1.07
	1	39.84	22.15	1.79	46.17	35.00	1.61
	2	36.76	18.31	2.00	76.12	34.61	2.10
	5	32.71	10.81	3.02	32.71	10.81	3.02
Polypropylene	0	14.48	61.33	0.23	14.48	61.33	0.23
	1	12.77	80.00	0.159	19.36	133.00	0.14
	2	15.50	90.67	0.178	21.08	157.21	0.13
	5	6.73	40.00	0.168	20.27	26.67	0.76
Polystyrene	0	17.50	13.53	1.29	17.50	13.53	1.29
	1	18.80	25.00	0.75	21.15	39.51	0.53
	2	18.45	19.75	0.93	22.34	42.15	0.53
	5	16.50	8.17	2.03	17.72	12.34	1.43

**Table 3:** TGA data of neat SBP and different nanoclays [78].

Organoclay wt.%	T_0_ (°C)	T_5_ (°C)	T_10_ (°C)
Neat SBP	249	397	454
1%	285	422	469
3%	272	414	461
5%	254	387	433

**Table 4:** Thermal properties of pure PECA and PECA–modified Mt composites with different Wt.% [79].

Wt.% of Modified MMT	T_10_ (°C)	T_50_ (°C)	Char Residue at 350 °C
Pure PECA	160	210	0.94
1%	169	221	1.39
3%	162	227	1.24
5%	166	222	4.37
7%	167	224	3.85

**Table 5:** Summary studies on water permeability of polymer–clay nanocomposites.

Polymer	Filler	Clay Content	Maximum Reduction in Permeability%	Refs
Polylactic Acid PLA	Closite 30B	5 wt%	60%	[86]
		5 wt%	58%	[87]
		15 wt%	95%	[87]
	MMT	6 wt%	43%	[88]
Polystyrene PS	MMT	6 wt%	70%	[89]
		10 wt%	54%	[90]
Polyurethane PU	MMT	40 wt%	90%	[91]
Polyacrylamide PA6	MMT	4 vol%	30%	[92]

## Data Availability

Data are contained within the article.
